# Polyethylene Glycol-Chitosan Oligosaccharide-Coated Superparamagnetic Iron Oxide Nanoparticles: A Novel Drug Delivery System for Curcumin Diglutaric Acid

**DOI:** 10.3390/biom10010073

**Published:** 2020-01-02

**Authors:** Feuangthit Niyamissara Sorasitthiyanukarn, Chawanphat Muangnoi, Wuttinont Thaweesest, Pahweenvaj Ratnatilaka Na Bhuket, Pongsakorn Jantaratana, Pornchai Rojsitthisak, Pranee Rojsitthisak

**Affiliations:** 1 Metallurgy and Materials Science Research Institute, Chulalongkorn University, Bangkok 10330, Thailand; Feuangthit.n@chula.ac.th; 2 Natural Products for Ageing and Chronic Diseases Research Unit, Chulalongkorn University, Bangkok 10330, Thailand; chawanphat.mua@gmail.com (C.M.); wuttinont.t@student.chula.ac.th (W.T.); bpahweenvaj@gmail.com (P.R.N.B.); pornchai.r@chula.ac.th (P.R.); 3 Institute of Nutrition, Mahidol University, Nakhon Pathom 73170, Thailand; 4 Department of Physics, Faculty of Science, Kasetsart University, Bangkok 10900, Thailand; fscipsj@ku.ac.th; 5 Department of Food and Pharmaceutical Chemistry, Faculty of Pharmaceutical Sciences, Chulalongkorn University, Bangkok 10330, Thailand

**Keywords:** polyethylene glycol, chitosan oligosaccharide, superparamagnetic iron oxide nanoparticles, curcumin diglutaric acid, drug delivery systems

## Abstract

Curcumin diglutaric acid-loaded polyethylene glycol-chitosan oligosaccharide-coated superparamagnetic iron oxide nanoparticles (CG-PEG-CSO-SPIONs) were fabricated by co-precipitation and optimized using a Box–Behnken statistical design in order to achieve the minimum size, optimal zeta potential (≥ ±20 mV), and maximum loading efficiency and capacity. The results demonstrated that CG-PEG-CSO-SPIONs prepared under the optimal condition were almost spherical in shape with a smooth surface, a diameter of 130 nm, zeta potential of 30.6 mV, loading efficiency of 83.3%, and loading capacity of 8.3%. The vibrating sample magnetometer results of the optimized CG-PEG-CSO-SPIONs showed a superparamagnetic behavior. Fourier transform infrared spectroscopy and X-ray diffraction analyses indicated that the CG physically interacted with PEG-CSO-SPIONs. In addition, the CG-PEG-CSO-SPIONs could be stored dry for up to 12 weeks or in aqueous solution for up to 4 days at either 4 °C or 25 °C with no loss of stability. The CG-PEG-CSO-SPIONs exhibited a sustained release profile up to 72 h under simulated physiological (pH 7.4) and tumor extracellular (pH 5.5) environments. Furthermore, the CG-PEG-CSO-SPIONs showed little non-specific protein binding in the simulated physiological environment. The CG-PEG-CSO-SPIONs enhanced the cellular uptake and cytotoxicity of CG against human colorectal adenocarcinoma HT-29 cells compared to free CG, and more CG was delivered to the cells after applying an external magnetic field. The overall results suggest that PEG-CSO-SPIONs have potential to be used as a novel drug delivery system for CG.

## 1. Introduction

Superparamagnetic iron oxide nanoparticles (SPIONs) have received increasing attention in drug delivery applications because they can be fabricated, characterized, and modified for their functional properties [1,2,3,4]. For instance, drug-loaded SPIONs can be delivered into targeted cancer cells through systemic or localized blood circulation by applying an external localized magnetic field gradient and, consequently, the payload drug is released and acts locally [5]. In general, SPIONs easily agglomerate due to the strong magnetic dipole-dipole attractions between particles, which results in a low stability and inbiocompatibility [6]. To overcome these limitations, several approaches have been studied, such as surface modification with various polymers [7,8]. Although surface modification of SPIONs can successfully be achieved by both synthetic and natural polymers, the latter, and especially polysaccharides, are more attractive due to their biodegradability, biocompatibility, and safe to human health status [9,10,11,12,13].

Among the various natural polymers, chitosan has been extensively studied due to its biodegradability, biocompatibility and hydrophilicity. However, chitosan is not soluble in water at neutral or alkaline conditions, making it difficult for biological applications [14]. Therefore, chitosan oligosaccharide (CSO) was chosen for this study due to its solubility over a wide pH range from acid to alkaline conditions making it more suitable for various biological applications [14,15]. Many studies have reported that coating SPIONs with CSO enhanced their solubility in an aqueous environment, biocompatibility and stability with a reduction of their toxicity [16,17].

Another interesting polymer is poly (ethylene glycol) (PEG), which has been approved by US-FDA for human oral, intravenous and dermal applications. Previous research has demonstrated that coating SPIONs with PEG could reduce the reticuloendothelial system clearance, toxicity and enzymatic degradation and increase the water solubility and stability of SPIONs, as well as prolonging their circulation half-life in vivo [18,19,20]. Thus, SPIONs coated with CSO and PEG could be a potential novel carrier for drug delivery applications [16,21,22].

**Figure 1:**
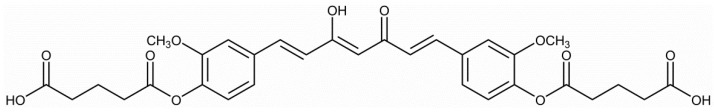
Chemical structure of curcumin diglutaric acid (CG).

Curcumin diglutaric acid (CG) (Figure 1) is a prodrug of curcumin and is synthesized by conjugation of curcumin with glutaric acid via an ester linkage. It shows better antinociceptive and anticancer properties with higher water solubility compared to traditional curcumin [23]. However, CG degrades in an aqueous environment, especially in an alkaline pH, resulting in poor oral bioavailability [23]. One possibility to overcome this limitation is to incorporate CG in suitable drug carriers, like SPIONs stabilized with PEG-CSO.

Therefore, this study was undertaken to examine the parameters affecting the characteristics of Curcumin diglutaric acid-loaded polyethylene glycol-chitosan oligosaccharide-coated superparamagnetic iron oxide nanoparticles (CG-PEG-CSO-SPIONs) and to optimize the formulation using a Box-Behnken statistical design (BBD) in order to achieve the minimum particle size, optimal zeta potential (≥ ±20 mV), and maximum loading efficiency (LE) and loading capacity (LC) of CG-PEG-CSO-SPIONs. The optimized CG-PEG-CSO-SPIONs were then investigated for the kinetic release, cellular uptake, and cytotoxicity of CG, including with magnetic targeting, using the human colorectal adenocarcinoma (HT-29) cell lines.

## 2. Materials and Methods

### 2.1. Chemicals and Reagents

The CG was synthesized as previously described [23]. The CSO (Mw 1500–1600 Da, degree of deacetylation ≥98%) was purchased from Kono Chem Co., Ltd. (Xi’an, China), while PEG (Mw 2000 Da) and cetyltrimethylammonium bromide (CTAB) were purchased from Acros Organics^TM^ (Morris Plains, New Jersey, USA). Ferric chloride hexahydrate (FeCl_3_·6H_2_O; purity > 99%), ferrous chloride tetrahydrate (FeCl_2_·4H_2_O; purity > 99%) and 3-(4,5-dimethylthiazole-2-yl)2,5-diphenyl tetrazolium bromide (MTT) were purchased from Sigma-Aldrich (St. Louis, MO, USA). Ammonium hydroxide solution (30% (*v*/*v*) NH_3_ in H_2_O) was from Carlo-Erba reagents (Val de Reuil, France). Ultrapure water was obtained using a Milli-Q^®^ water purifier (Millipore, Molsheim, France). Other reagents were all of analytical grade and used without further purification.

### 2.2. Cell Culture

The cytotoxicity of nanoparticles (NPs) were tested in vitro against the HT-29 human colorectal adenocarcinoma cell line (ATCC, Manassas, VA, USA). Cells were grown in complete medium (CM), comprised of Dulbecco’s modified eagle medium (Invitrogen, Grand Island, NY, USA) supplemented with 10% (*v*/*v*) fetal bovine serum (FBS; Hyclon, MA, USA) and 1% (*v*/*v*) penicillin/streptomycin (Sigma). The cells were maintained in culture at 37 °C in a humidified atmosphere of 95:5 (*v*/*v*) air: CO_2_.

### 2.3. Fabrication of Core-Shell NPs

#### 2.3.1. Fabrication of SPIONs

The SPIONs were fabricated by co-precipitation as previously described [24] with modifications. Typically, 10 mL of a mixed solution of 0.6 g FeCl_2_·4H_2_O and 0.82 g FeCl_3_·6H_2_O was prepared at room temperature and 2.5 mL of ammonium hydroxide solution (30%) was then added into the mixture with vigorous magnetic stirring at 1000 rpm until the solution reached pH 10. After stirring for 30 min, the mixed solution was heated at 80 °C for 45 min maintaining vigorous stirring. The precipitate was collected by magnetic separation and washed several times with ethanol and water to remove excess ammonia. The resulting SPIONs were lyophilized at −50 °C for 24 h and stored in a desiccator for further use.

#### 2.3.2. Coating SPIONs with CSO and PEG

The SPIONs were then coated with CSO and PEG as previously reported [25] with some modifications. In brief, powdered SPIONs (250 mg) were dispersed into 400 mL of water containing various concentrations of CTAB. Next, 100 mL of CSO solution (various concentrations) in 1% (*w*/*v*) acetic acid was added and stirred vigorously for 1 h at room temperature. A 100 mL of PEG solution (various concentrations) in water was then added into the mixed solution and further stirred for 1 h. The obtained PEG-CSO-SPIONs were harvested and stored as previously described in Section 2.3.1.

#### 2.3.3. Loading of CG onto the PEG-CSO-SPIONs

The CG was loaded onto PEG-CSO-SPIONs by a soaking method as previously described [25] with modifications. Typically, powdered PEG-CSO-SPIONs (250 mg) were added into the desired concentration of CG solution dissolved in ethanol (5 mL) and mixed thoroughly on an orbital shaker at 150 rpm at room temperature for 3 h. Finally, the resulting CG-PEG-CSO-SPIONs were collected and stored as previously described in Section 2.3.1.

### 2.4. Experimental Design

#### 2.4.1. Factorial Design for Preparation of CG-PEG-CSO-SPIONs

A Plackett-Burman design (PBD), provided by the Design Expert^®^ software version 10 (trial version, Minneapolis, MN, USA), was used for screening the significant factors and evaluating their interaction with the responses. The preliminary screening experiments with five factors (CTAB, CSO, and PEG concentrations, molecular weight of PEG, and CG concentration) at high (+) and low (−) levels, and the responses (particle size, zeta potential, LE, and LC) are summarized in Table 1 and Table 2, respectively. A Pareto chart was generated using the PBD in order to determine the statistically significant factors that were potentially the crucial main effects.

#### 2.4.2. Graphical Drawing, Optimization, and Statistical Analysis

Based on the statistical results obtained from the PBD analysis, the CTAB (X_1_), CSO (X_2_), and PEG (X_3_) concentrations were recognized as the statistically significant factors over the range of 2.5–7.5 mg/mL, 0.1–0.3 mg/mL, and 0.1–0.3 mg/mL, respectively. A BBD was then used to investigate the effect of each factor on the response, based on the PBD preliminary screening. A total of 15 experimental runs were generated, and all experiments were performed in triplicate. ANOVA was used to identify the fitness of the model and the significant factors affecting the responses. The fabrication parameters of the CG-PEG-CSO-SPIONs are shown in Table 3 and Table 4, respectively.

### 2.5. Characterization of the NPs

The average particle size, as the hydrodynamic diameter, and zeta potential were measured using a Zetasizer, Nano-ZS (Malvern Instruments, Malvern, UK). The morphology was visualized using transmission electron microscopy (TEM; model H-9500, Hitachi High Technology America Inc., Pleasanton, CA, USA), while the interaction between CG and PEG-CSO-SPIONs was evaluated using Fourier transform infrared spectroscopy (FT-IR) with a FTIR-ALPHA (Bruker, Malvern, PA, USA) instrument. The crystal structures were investigated by X-ray diffraction (XRD), which was performed in a PW3710 X-ray diffractometer (Philips, Eindhoven, The Netherlands) using the Kα line of Cu as the radiation source (1.542 Å) and operated at 40 kV and 30 mA, respectively. The magnetization property was measured using a vibrating sample magnetometer (VSM; Lake Shore Cryotronics, Inc., OH, USA). The equilibrium times for the release of CG from the CG-PEG-CSO-SPIONs in the acidic and alkaline RM were 24 and 48 h, respectively. To determine the drug LE and LC, the CG-PEG-CSO-SPIONs were magnetically separated from the CG solution after soaking for 3 h and the remaining CG present in the solution was measured using a Cary-60 UV–VIS spectrophotometer (Agilent Technologies, CA, USA) at a wavelength of 402 nm. The LE and LC were then calculated using Equations (1) and (2), respectively:

LE (%) = [(W_t_ − W_s_)/W_t_] × 100 (1)

LC (%) = [(W_t_ − W_s_)/W_np_] × 100 (2)

where W_t_ is the amount of CG initially added in the formulation, W_s_ is the amount of residual (non-bound) CG in the solution, and W_np_ is the dry mass of the CG-PEG-CSO-SPIONs.

### 2.6. In Vitro Release of CG: Kinetics

The in vitro release of CG from CG-PEG-CSO-SPIONs was evaluated using dialysis at physiological (pH = 7.4) and tumor extracellular (pH = 5.5) conditions. For this, 250 mg of dried CG-PEG-CSO-SPIONs or free CG at the equivalent concentration was dispersed in 10 mL of 1:1 (*v*/*v*) phosphate buffered saline (PBS; pH 5.5 or 7.4): ethanol as the releasing medium (RM) and loaded into a separated dialysis bag (Mw cutoff 35 kDa, Fisher Scientific, PA, USA). The ethanol was used to enhance the solubility of CG in the RM. The bags were then separately suspended in 90 mL of RM at 37 °C with stirring at 150 rpm. At the indicated time intervals, a 2-mL sample was withdrawn and replaced with an equal volume of fresh RM. Samples were analyzed using a UV–VIS spectrophotometer at 402 nm to determine the CG concentration. All experiments were performed in triplicate.

The mechanism of CG release from the different NPs was investigated using zero-order, first-order, Higuchi, and Korsmeyer-peppas mathematical models in the DDSolver Excel sheet software application (China Pharmaceutical University, Nanjing, China). The criteria for selection of the best fit model were an R^2^_adjusted_ close to 1 with a high model selection criteria (MSC) value and low Akaike information criterion (AIC) [26,27]. The Korsmeyer-peppas model has been extensively used in several studies for the analysis of drug release kinetics in matrix systems, where Fickian diffusion is represented by n ≤ 0.45, anomalous transport by 0.45 < n < 0.89, and case II transport (zero-order release) by n = 0.89 [28].

### 2.7. In Vitro Protein Binding

The protein binding of CG-PEG-CSO-SPIONs was investigated as previously described [21]. In brief, 5 mg of dried CG-PEG-CSO-SPIONs were incubated with various bovine serum albumen (BSA) concentrations (5 mL) in an orbital shaker at 37 °C for 6 h. The BSA solution was used as a control. The sample was then centrifuged at 16,000× *g* at 4 °C for 10 min, and the BSA concentration found in the supernatant was analyzed using UV–VIS spectroscopy at 595 nm following the Bradford method. The protein binding concentration was deduced from the mass balance of proteins. In addition, to confirm the absence of non-specific protein binding on the surface of the NPs, any change in the particle size after incubation in the BSA solution was ascertained by TEM analysis as previously described [29] with some modifications. Briefly, 20 µL of BSA solution was added to the CG-PEG-CSO-SPION suspension (1 mg/mL) and stirred for 2 h at 25 °C. The BSA-bound CG-PEG-CSO-SPIONP pellet was then magnetically separated from the residual BSA solution and re-dispersed in water prior to measuring the particle size using TEM.

### 2.8. Storage Stability of CG-PEG-CSO-SPIONs

The storage stability of the CG-PEG-CSO-SPIONs was investigated in two different time periods (short- and long-time storage) as previously described [30] with modifications. In this study, the stability of the CG-PEG-CSO-SPIONs was determined on the physicochemical properties of nanoparticles during the short- and long-term storage. The amount of CG in the nanoparticles during the storage stability study was not determined. However, in our preliminary experiment, the amount of CG in CG-PEG-CSO-SPIONs was not significantly changed after seven days of storage. For the short-time aqueous storage stability, the optimized powdered CG-PEG-CSO-SPIONs were dispersed in water and stored at either 4 °C or 25 °C. The temperatures at 4 °C and 25 °C were chosen for the stability study of CG-PEG-CSO-SPIONs according to the recommendation of the ICH guideline [31]. At predetermined times (1, 2, 3, and 4 days), the physico-chemical properties, including particle size (hydrodynamic diameter) and zeta potential, were investigated. For the long-time dry storage stability, the powdered CG-PEG-CSO-SPIONs were stored in amber vial bottles at 4 °C and 25 °C and at the predetermined times (1, 2, 4, 6, 8, 10, and 12 weeks), the samples were re-dispersed in water and then characterized as described above.

### 2.9. Cell Experiments

#### 2.9.1. Cytotoxicity Studies

The cell viability was estimated using the MTT assay as previously reported [32] with modifications. Briefly, HT-29 cells were seeded in CM at 1 × 10^4^ cells/well in 96-well plates and incubated at 37 °C with 5% CO_2_ for 24 h. Cells were then washed with serum-free CM and incubated with the sample suspensions (free CG, SPIONs, PEG-CSO-SPIONs, and CG-PEG-CSO-SPIONs) in CM for 24 h. The MTT solution (0.5 mg/mL in PBS) was then added and incubated at 37 °C for 4 h. After removal of the culture media, cells were washed with PBS, suspended in 200 µL dimethylsulphoxide (DMSO) to dissolve the formazan crystals and the optical density at 540 nm was measured using a microplate reader (CLARIOstar, BMG Labtech, Germany). Each experiment was performed in triplicate and the data are presented as the mean ± standard deviation (SD). Cell viability was calculated using Equation (3):

Cell viability (%) = (N_t_/N_c_) × 100 (3)

where N_t_ and N_c_ are the optical densities of the treated and untreated (control) cells, respectively.

#### 2.9.2. In Vitro Cellular Uptake

In vitro cellular uptake of free CG and the CG from the CG-PEG-CSO-SPIONs were investigated by confocal laser scanning microscopy (CLSM) as previously reported [32] with modifications. In brief, HT-29 cells were cultured in CM and treated with the respective sample as described in Section 2.9.1. After incubation for 24 h, CSLM cell images were captured using a D-ECLIPSE C1 confocal laser scanning microscope (Nikon, Shinagawa, Japan) at excitation and emission wavelengths of 400 nm and 470 nm, respectively. The extent of cellular uptake was expressed as the fluorescence intensity associated with the CG-PEG-CSO-SPIONs compared to that associated with the free CG solution or blank NPs (PEG-CSO-SPIONs).

#### 2.9.3. Magnetic Targeting Delivery Assay

In this study, an external magnet was used to enhance the localized delivery and hence cytotoxicity of the CG-PEG-CSO-SPIONs in cancer cells as previously described [29] with some modifications. Briefly, HT-29 cells were cultured and treated with free CG or CG-PEG-CSO-SPIONs with the same protocol as previously described (Section 2.9.2 and [29]), but the external neodymium magnet was placed under the cell culture plate during incubation of the cells with the respective sample. To further affirm the magnetic targeting of CG-PEG-CSO-SPIONs, the in vitro cellular uptake was evaluated by CLSM of cells treated with the respective sample with and without the applied external magnetic field.

### 2.10. Statistical Analysis

The data was processed using Microsoft Excel 2016 (Microsoft Corporation, Redmond, WA, USA) and are shown as the mean ± SD. Statistical analyses were performed using a two-sampled variance F-test, accepting significance at the *p* < 0.05 level.

## 3. Results and Discussion

### 3.1. Optimization of the CG-PEG-CSO-SPIONs by PBD and BBD

**Figure 2:**
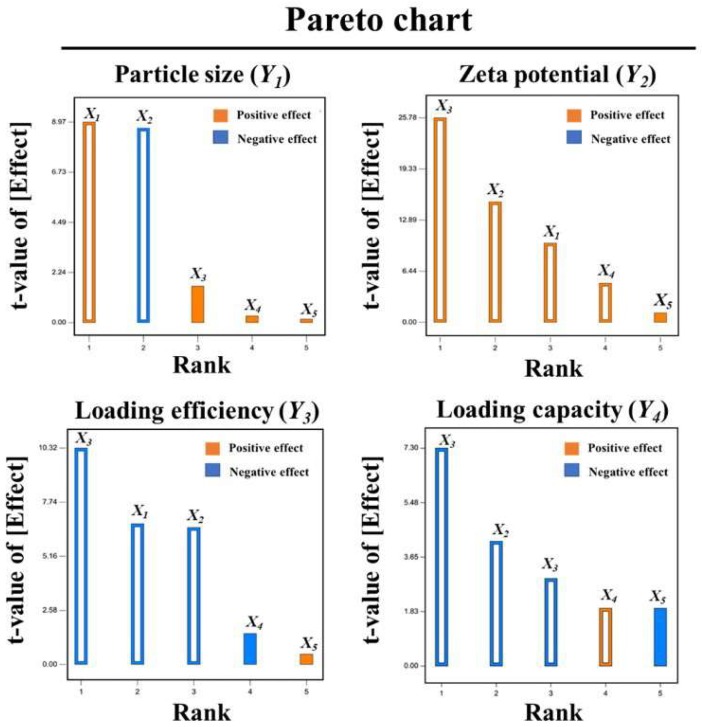
Plackett-Burman: Pareto charts revealing the significance of the factors on the determined responses. Significance of the given factor (X_1–5_) on the particle size (Y_1_), zeta potential (Y_2_), LE (Y_3_), and LC (Y_2_). The blue and orange colors indicated the negative and positive effects, respectively. The transparency inside both blue and orange demonstrated the mainly significant effect at *p* < 0.05.

Based on preliminary screening experiments using PBD, 15 experimental runs were generated from five factors, four responses, and two levels with three center points (at intermediate level), as shown in Table 1 and Table 2. To investigate the significant factors affecting the responses, statistical evaluation was performed through ANOVA and a Pareto chart (Figure 2). The results reveal that CTAB (X_1_), CSO (X_2_), and PEG (X_3_) concentrations are the main significant effect to four responses in comparison with molecular weight of PEG (X_4_) and CG concentration (X_5_). Thus, the factors X_1_, X_2_, and X_3_ were selected as crucial affecting factors for further optimization by a three-factor, three-level, and three-center point BBD. Quadratic regression equations and three-dimensional response surface plots (3D-RSPs) were generated for each response. Compared with other models including the linear and interaction (2FI) models, statistically significant *p*-values indicated a goodness of fit for all responses, affirmed by the statistical analysis of the quadratic model (Table 5).

The particle size (Y_1_) and zeta potential (Y_2_) of CG-PEG-CSO-SPIONs, according to the BBD analysis, ranged between 92 ± 32 nm and 362 ± 19 nm and from 20.2 ± 2.3 mV to 33.1 ± 1.6 mV, respectively, (Table 4). The quadratic regression equation, which represents the relationship between the factors to the particle size and zeta potential, is shown in Equations (4) and (5), respectively:

Particle size (Y_1_) = 188.92 + 75.49X_1_ + 46.88X_2_ + 59.72X_3_ + 11.04X_1_X_2_ + 16.79X_1_X_3_ + 11.25X_2_X_3_ + 7.49X_1_^2^ + 16.95X_2_^2^ + 12.58X_3_^2^ (4)

Zeta potential (Y_2_) = 29.37 + 4.14X_1_ + 2.37X_2_ + 0.71X_3_ − 1.32X_1_X_2_ − 0.60X_1_X_3_ + 0.53X_2_X_3_ − 2.03X_1_^2^ + 0.64X_2_^2^ − 1.48X_3_^2^ (5)

The particle size and zeta potential of the CG-PEG-CSO-SPIONs were mainly affected by the CTAB concentration (X_1_) (*p* < 0.05), which had a positive effect on both responses. For instance, the particle size increased with increasing CTAB concentrations, which might be due to the increased level of CTAB adsorbed onto the surface of the NPs. Likewise, the zeta potential of the CG-PEG-CSO-SPIONs increased as the CTAB concentration increased (*p* < 0.05), presumably due to the increased formation of positively-charged micelles from the positively-charged CTAB on the surface of the negatively charged NPs resulting in charge neutralization.

**Figure 3:**
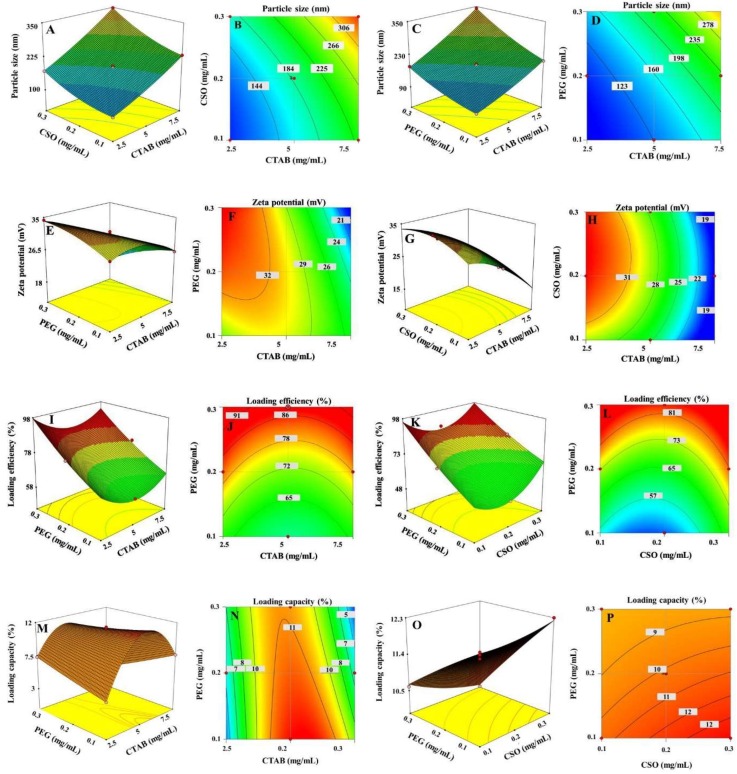
The 3D-RSPs and contour plots showing the effects of the CTAB (X_1_), CSO (X_2_), and PEG (X_3_) concentrations on the responses of the (**A**–**D**) particle size (Y_1_), (**E**–**H**) zeta potential (Y_2_), (**I**–**L**) LE (Y_3_), and (**M**–**P**) LC (Y_4_).

The 3D-RSPs and contour plots that were used to evaluate the effects of the factors on the particle size and zeta potential are shown in Figure 3. Contour plots indicated that the minimum particle size was obtained at the low level of all three factors, whereas the maximum zeta potential was obtained at the high level of CTAB and PEG and medium level of CSO. The ANOVA analysis of the particle size and zeta potential are summarized in Table 5, and indicated that the data fitted well with the models for size and zeta potential (R^2^ and R^2^_predicted_ values close to 1).

The LE (Y_3_) and LC (Y_4_) of CG-PEG-CSO-SPIONs ranged from 43.5 ± 4.6 to 86.4 ± 3.2% and from 3.1 ± 1.1 to 12.3 ± 1.3%, respectively, (Table 4). The effect of the PEG concentration (X_3_) was more remarkable than that of the CTAB and CSO concentrations, with the quadratic regression equations for both responses shown in Equations (6) and (7), respectively:

LE (Y_3_) = 53.73 − 2.25X_1_ + 4.57X_2_ + 16.98X_3_ + 0.93X_1_X_2_ − 0.52X_1_X_3_ − 2.68X_2_X_3_ + 8.60X_1_^2^ + 12.40X_2_^2^ + 2.10X_3_^2^ (6)

LC (Y_4_) = 11.26 − 0.36X_1_ + 0.28X_2_ − 0.52X_3_ + 0.71X_1_X_2_ − 1.78X_1_X_3_ − 0.18X_2_X_3_ − 5.59X_1_^2^ − 0.064X_2_^2^ + 0.072X_3_^2^ (7)

The 3D-RSPs and contour plots, as shown in Figure 3, revealed that at a high PEG concentration and low CTAB concentration, the LE and LC were increased by up to 86.4 ± 3.2% and 7.7 ± 2.8%, respectively. Increasing the CTAB concentration decreased the LE and LC to 43.5 ± 4.6% and 3.1 ± 1.1%, respectively. The contour plots suggested that the maximum LE was obtained at a high PEG concentration, medium CSO concentration and low CTAB concentration, respectively, whereas the maximum LC was obtained at a medium concentration of all three factors. Therefore, it was concluded that the LE and LC were enhanced with increasing PEG and CSO concentrations due to the strong interaction between the drug and polymer shell coated on the surface of the NPs, which increased the chance of the drug being adsorbed onto the surface of the NPs [24]. Furthermore, a high CTAB concentration led to increased micelle formation at the surface of the SPIONs. and so an increased particle size with a decreased specific area, which resulted in a lower drug LE and LC. The ANOVA analysis of the LE and LC is summarized in Table 5. The R^2^ value of LE and LC was 0.9919 and 0.9992, respectively, while the R^2^_predicted_ value was 0.8856 and 0.9959, respectively. All of the ANOVA analysis results were in reasonable agreement and so the design space could be navigated.

In order to obtain an improved formulation with a promising particle size, RSM optimization of the zeta potential, LE and LC was employed. Numerical optimization applying a desirability function was used to identify the optimum conditions for obtaining a minimum particle size, high zeta potential of ≥ ±20 mV and maximum LE and LC, and is shown in Table 6. The optimal conditions were defined as 2.8 mg/mL CTAB, 0.1 mg/mL CSO, and 0.26 mg/mL PEG, giving a desirability value of 0.96. The particle size, zeta potential, LE and LC were predicted as 133 nm, 30.6 mV, 83.3%, and 8.3%, respectively, (Table 6), which was further validated by comparison with the observed results. According to Table 6, the LC of the optimized CG-PEG-CSO-SPIONs was found to be 7.94%, indicating that the amount of CG at 7.94 g was contained in 100 g of dried CG-PEG-CSO-SPIONs. The relatively low percentage of error (<10%) suggested that the model was suitable for the optimization of CG-PEG-CSO-SPIONs.

### 3.2. Characterization of the NPs

**Figure 4:**
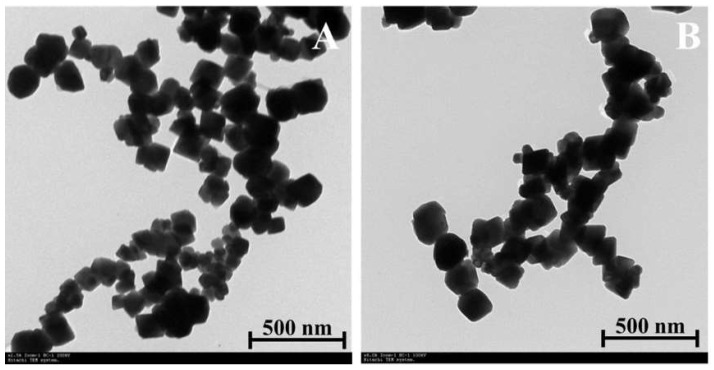
Representative TEM images (8000× magnification) of (**A**) SPIONs and (**B**) CG-PEG-CSO-SPIONs.

The size, morphology and particle uniformity of the SPIONs and CG-PEG-CSO-SPIONs fabricated under the optimal condition were investigated by TEM (Figure 4). The obtained SPIONs and CG-PEG-CSO-SPIONs had a small size of around 100 nm in diameter with smooth surface and narrow size distribution.

**Figure 5:**
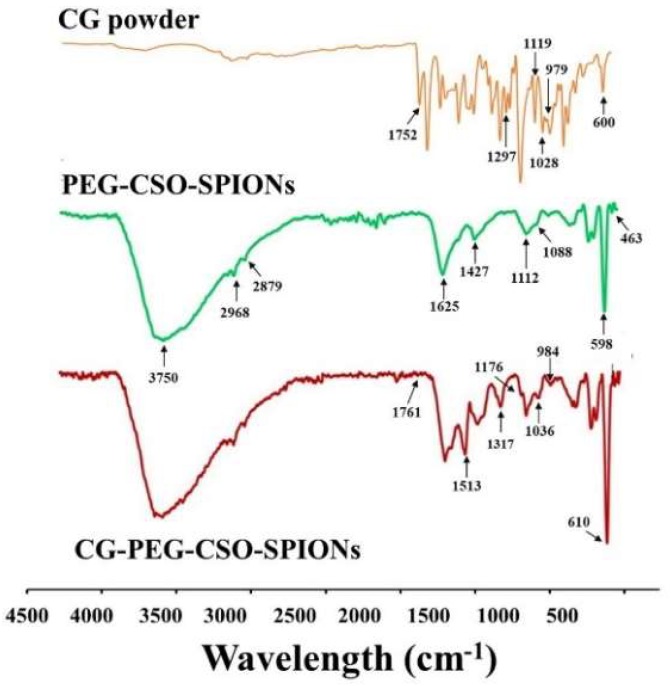
Representative FT-IR spectra of the CG powder; PEG-CSO-SPIONs and CG-PEG-CSO-SPIONs.

The overlay FT-IR spectra of the CG powder, PEG-CSO-SPIONs and CDG-PEG-CSO-SPIONs are shown in Figure 5. The spectrum of the CG powder showed its characteristic peaks at 1752 cm^−1^ (C=O stretching vibration of carboxylate ester), 1508 cm^−1^ (C=C vibrations), 1297 cm^−1^ (C-O stretching of carboxylic acid), 1119 cm^−1^ (aromatic C-C-H bending), 1028 cm^−1^ (symmetrical and asymmetrical C-O-C stretching vibration), 979 cm^−1^ (benzoate *trans*-CH vibration), and 600 cm^−1^ (*cis*-CH vibration of an aromatic ring). In the case of the PEG-CSO-SPIONs and SPIONs, they showed only two characteristic peaks at 598 cm^−1^ and 463 cm^−1^ (Fe-O vibration). However, the characteristic PEG peaks at 2879 cm^−1^ (C-H stretching of alkanes) and 1112 cm^−1^ (C-O-C of ether) and the characteristic CSO peaks at 3750 cm^−1^ (OH stretching vibration), 2968 cm^−1^ (C-H stretching), 1625 cm^−1^ (Amide I), and 1088 cm^−1^ (C=O), were also observed in the spectrum of PEG-CSO-SPIONs indicating the coating of PEG and CSO on the surface of the SPIONs. In addition, most of characteristic peaks of CG were found in the spectrum of the CG-PEG-CSO-SPIONs, confirming the physical interaction of CG with the NP matrix and also suggesting that the CG structure and its integrity were not affected by PEG-CSO-SPIONs.

**Figure 6:**
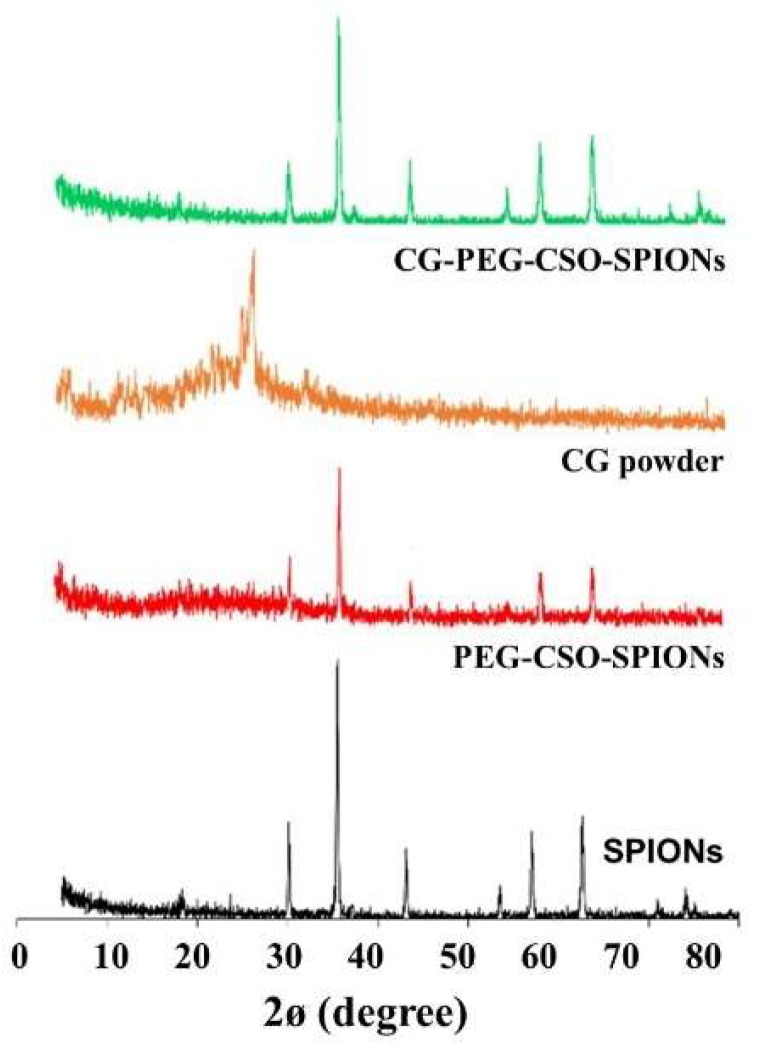
Representative XRD patterns of SPIONs, PEG-CSO-SPIONs; CG powder, and CG-PEG-CSO-SPIONs.

The XRD pattern of SPIONs, PEG-CSO-SPIONs, CG powder and CG-PEG-CSO-SPIONs are shown in Figure 6. The sharp XRD pattern of the SPIONs revealed its characteristic peaks at 30.2°, 35.5°, 43.1°, 53.6°, 57.1° and 62.6°, respectively, indicating the relatively high crystallinity of the SPIONs [25]. The crystalline structure of SPIONs was not changed after coating with PEG-CSO and loading with CG. Thus, the polymer coating and CG loading likely occurred at the surface of the SPOINs and did not affect their characteristics. In addition, the characteristic XRD patterns of the CG powder were not found in the pattern of the CG-PEG-CSO-SPIONs, which may indicate the amorphous state of a solid molecular dispersion or a solid solution inside the polymers coated on the surface of the SPIONPs after adsorption [33].

**Figure 7:**
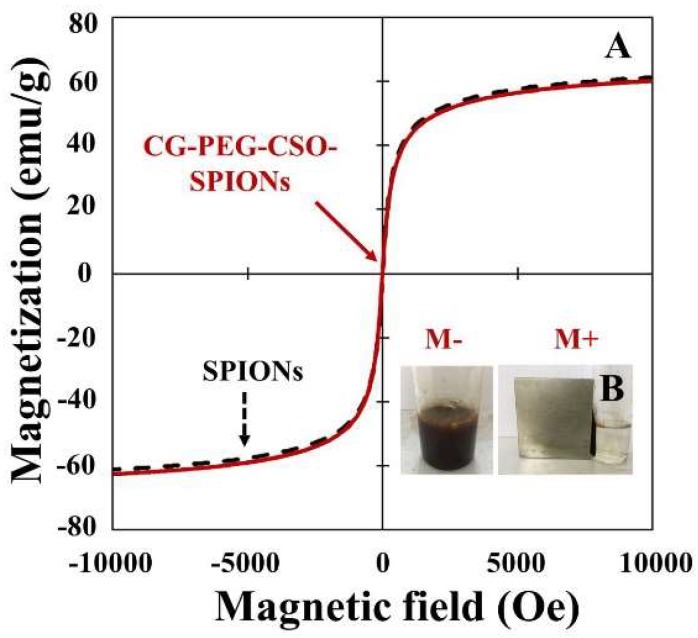
Representative VSM analysis showing the magnetic behavior of (**A**) SPIONs and CG-PEG-CSO-SPIONs and (**B**) the localization of CG-PEG-CSO-SPIONs near an external magnet. (M−) Without magnet; (M+) with magnet.

The use of field magnetization to aid the uptake of SPIONs and CG-PEG-CSO-SPIONs into HT-29 cells is summarized in Figure 7A. The magnetization of SPIONs was about 60 emu/g and was reduced to below 1% after PEG-CSO coating and CG loading. In the other words, the polymer coating and CG loading did not affect the magnetic characteristics of the SPIONs [34]. The saturated magnetization for SPIONs and CG-PEG-CSO-SPIONs (~60 emu/g) were lower than that of the bulk iron oxide particles (~92 emu/g) [35], and no hysteresis loop was observed, indicating the superparamagnetic behavior. Especially, the saturation remanence (M_s_) and coercivity (H_c_) values of the CG-PEG-CSO-SPIONs were about 0.52 emu/g and 4.11 Oe, respectively, which indicated a low residual magnetization was present when the external magnetic field was removed, and that a low intensity magnetic field is required to reduce the magnetization to zero. This may suggest that the CG-PEG-CSO-SPIONs can be separated and controlled by an external magnetic field (Figure 7B), which would be suitable for targeted drug delivery applications.

### 3.3. In Vitro CG Release Kinetics from the Optimized CG-PEG-CSO-SPIONs

**Figure 8:**
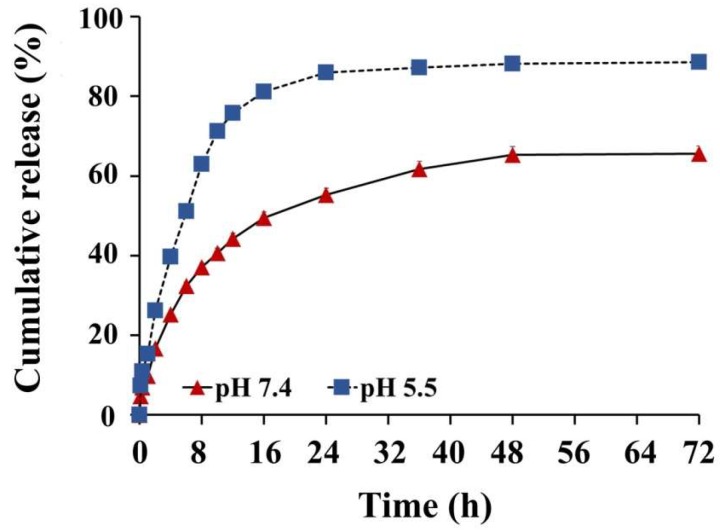
In vitro cumulative CG release from CG-PEG-CSO-SPIONs in the pH 5.5 and 7.4 RM over 72 h. Data are shown as the mean ± SD from three replications.

Figure 8 shows the cumulative release of CG from the optimized CG-PEG-CSO-SPIONs in the RM at pH 5.5 or 7.4. The fast release was observed at the first 8 h for both pH values due to the swelling of polymer layers on SPIONs, and consequently, the weakly adsorbed CG molecules on the surface of PEG-CSO-SPIONs were easily released. The CG was then slowly released from CG-PEG-CSO-SPIONs. Finally, a sustained release manner was observed with a faster release in the acidic RM comparing to the alkaline RM. This might reflect the higher solubility of CG and CSO in an acidic environment. The maximum drug release was observed at pH 5.5 and 7.4 was 89% and 66%, respectively.

The drug release profile was fitted to the kinetic models in the DDsolver software. Based on the best fit model selection criteria (highest R^2^_adjusted_ and AIC with lowest MSC), the CG release from CG-PEG-CSO-SPIONs best fitted with the Korsmeyer-Peppas model at both pH 5.5 (n = 0.30, R^2^_adjusted_ = 98%, AIC = 98.03, and MSC = 1.85) and pH 7.4 (n = 0.35, R^2^_adjusted_ = 97%, AIC = 88.81, and MSC = 2.80). In addition, in this study n was in the range of 0.30–0.35, which indicated that Fickian diffusion was the controlling factor for CG release from the NPs.

### 3.4. In Vitro Protein Binding

**Figure 9:**
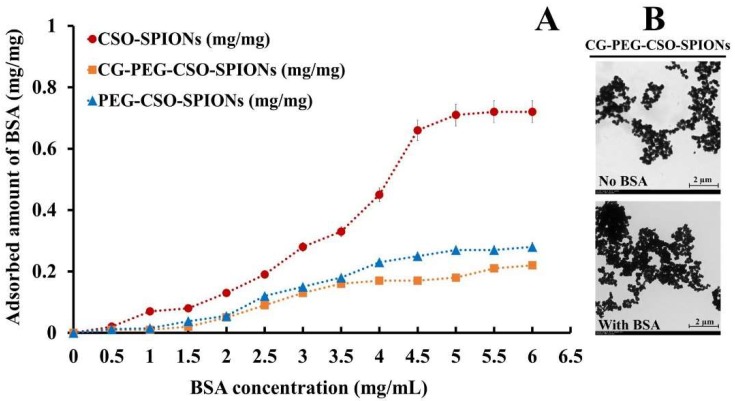
In vitro protein (BSA) binding onto the (**A**) CSO-SPIONs; PEG-CSO-SPIONs; CG-PEG-CSO-SPIONs, and (**B**) a representative TEM image of the CG-PEG-CSO-SPIONs before and after incubation with the BSA solution.

The plasma protein binding on the surface of nanoparticles is one of the key factors that affects biodistribution, biocompatibility, and therapeutic efficacy of drug-loaded nanoparticles. In addition, the protein binding has potentially serious consequences such as hemolysis, thrombosis, and embolization [36]. Therefore, not only is a low level of protein binding required in preclinical and human applications. In the present study, BSA was used as a model protein to investigate the non-specific protein binding level of the developed NPs. The binding capacity of BSA onto the surface of the CSO-SPIONs, PEG-CSO-SPIONs and CG-PEG-CSO-SPIONs is shown in Figure 9A. The BSA binding capacity of the CSO-SPIONs decreased significantly after coating with PEG (*p* < 0.05). Whereas it showed only a slight difference in BSA binding after loading CG onto the PEG-CSO-SPIONs. Furthermore, the highest amount of BSA bound onto the surface of CSO-SPIONs was approximately 2.9- and 3.2-fold higher than that on the PEG-CSO-SPIONs and CG-PEG-CSO-SPIONs, respectively. This phenomenon might be attributed to steric repulsion via the flexible PEG chains in solution that can then shield the CSO particles more efficiently from protein binding due to its electrostatic interaction [21]. In addition, no significant change in the aggregation of CG-PEG-CSO-SPIONs before and after incubation with the BSA solution was noted (Figure 9B). Although a slightly increased particle size of the NPs was observed after incubation in the BSA solution, which might be the bound layer of BSA on the surface of the NPs [29].

### 3.5. Storage Stability

**Figure 10:**
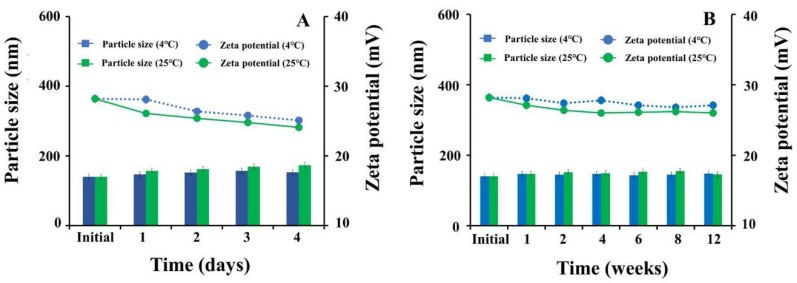
Particle size and zeta potential of CG-PEG-CSO-SPIONs after (**A**) four days of aqueous storage (short term stability) and (**B**) after 12 weeks of dry storage (long term stability) at 4 °C or 25 °C. Data are shown as the mean ± SD, derived from three replications.

The storage stability of CG-PEG-CSO-SPIONs was investigated at 4 °C and 25 °C for both the short-term aqueous storage of up to four days (Figure 10A) and the long-term dry storage up to 12 weeks (Figure 10B). No significant (*p* > 0.05) change in the particle size or zeta potential was found at either storage temperature or time, but rather the particle size and zeta potential were stable.

### 3.6. Cytotoxicity Studies

**Figure 11:**
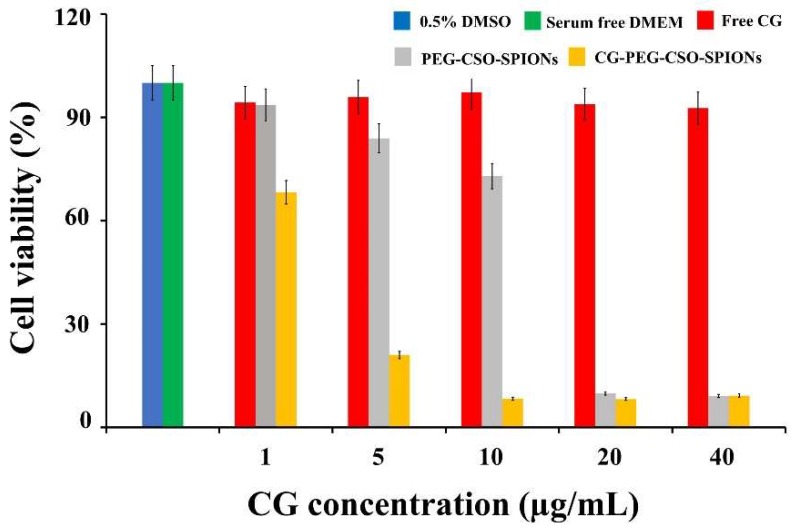
HT-29 cell viability after treatment with control (PEG-CSO-SPIONs), free CG, and CG-PEG-CSO-SPIONs for 24 h. Data are shown as the mean ± SD, derived from four trials.

The cytotoxicity of free CG, PEG-CSO-SPIONs and CG-PEG-CSO-SPIONs against HT-29 cells was examined. Serum-free CM and 0.5% (*v*/*v*) DMSO was used as the control for the NPs and free CG, respectively, and neither showed any significant cytotoxicity to HT-29 cells in all the assays. As shown in Figure 11, PEG-CSO-SPIONs showed a dose-dependent cytotoxicity on HT-29 cells, whereas the free CG solution showed no cytotoxicity on HT-29 cells. Interestingly, the cytotoxic effect of the PEG-CSO-SPIONs against HT-29 cells significantly (*p* < 0.05) increased after CG loading, which might be due to a synergistic effect of the magnetic NPs and CG on cell uptake. However, the toxicity of the PEG-CSO-SPIONs on HT-29 cells increased with increasing NP concentrations above 5 µg/mL (cell viability <80%), potentially due to the high release of iron ions in the intracellular space and in situ degradation [17].

The higher toxicity of PEG-CSO-SPIONs might result from the chemopreventive activity of CSO on HT-29 cells by increasing quinine reductase (QR), glutathione-S-transferase (GST) activities, glutathione (GSH) levels, and by inhibiting the ornithine decarboxylase (ODC) activity and COX-2 expression [37]. However, it was observed that the cytotoxicity activity of PEG-CSO-SPIONs against HT-29 cells was significantly increased by loading of CG (*p* < 0.05). For example, at 5 µg/mL of CG, the viability of HT-29 cells after treatment with the PEG-CSO-SPIONs was 84% compared to 21% for CG-PEG-CSO-SPIONs, reflecting the synergistic cytotoxicity of the PEG-CSO-SPIONs and CG. At CG concentrations up to 40 μg/mL, the cytotoxicity of CG-PEG-CSO-SPIONs was significantly higher than that of free CG (*p* < 0.05). For example, at 10 µg/mL of CG, the viability of HT-29 cells treated by free CG (97%) was 11-fold higher than those treated with CG-PEG-CSO-SPIONs (8.3%).

### 3.7. In Vitro Cellular Uptake of CG

**Figure 12:**
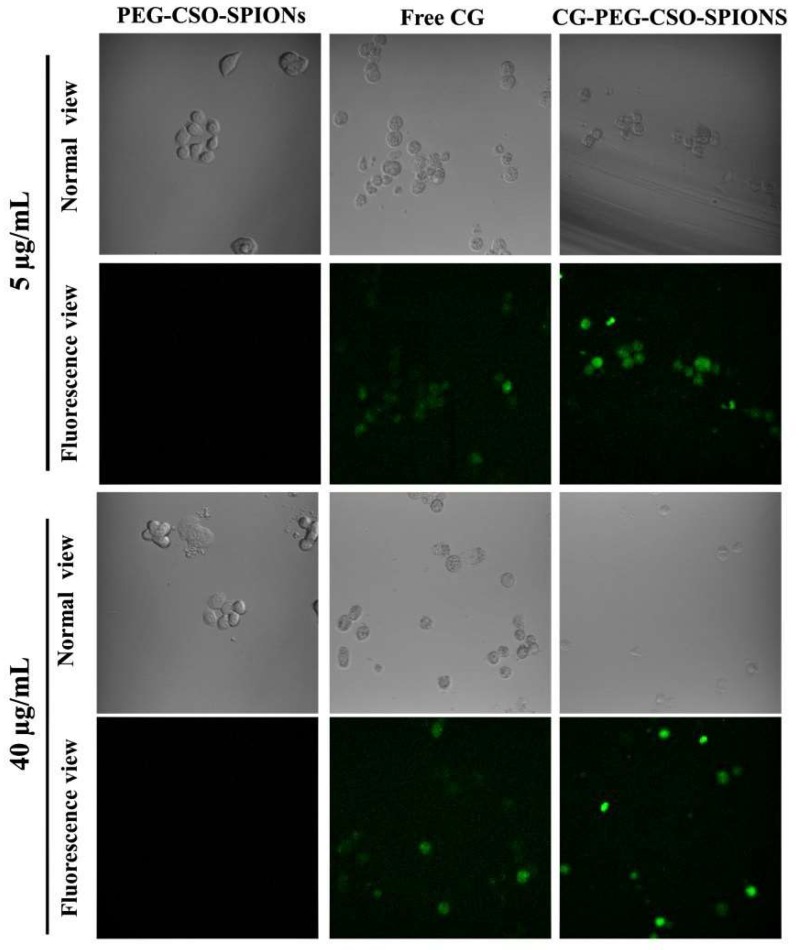
Representative CLSM images of CG uptake by HT-29 cells after 24 h of treatment with CG-PEG-CSO-SPIONs or free CG.

Enhanced cellular internalization or cellular uptake of drug loaded-NPs in cancer cells is typically an indication of a higher therapeutic index [29]. Thus, the present study determined the qualitative uptake of CG-PEG-CSO-SPIONs by HT-29 cells. Since the chemical structure of CG contains a fluorophore with maximum excitation and emission wavelengths of 400 and 470 nm, respectively, fluorescent dye staining is not required for visualization of the cellular uptake of free CG or CG-PEG-CSO-SPIONs. Rather, CLSM images can represent the cellular uptake of free CG and CG-PEG-CSO-SPIONs, and those at two different CG concentrations (5 ug/mL and 40 ug/mL) are shown in Figure 12. The cellular uptake of CG and CG-loaded nanoparticles was determined based on the fluorophore of CG using CLSM. The blank nanoparticles were used as a control and no fluorescent signal was observed. However, the fluorescent signals were observed for the cellular uptake of CG and CG-loaded nanoparticles, suggesting the cellular internalization of CG and CG-loaded NPs. The higher intensity of the CG-loaded nanoparticles comparing to free CG suggests that the nanoparticles can enhance the cellular uptake of CG. The green fluorescence intensity in the HT-29 cells treated with free CG or CG-PEG-CSO-SPIONs increased as the loaded CG concentration increased from 5 ug/mL to 40 ug/mL, whereas the blank NPs did not give any fluorescent signal. Thus, the cellular uptake of free CG and CG-PEG-CSO-SPIONs by HT-29 cells was concentration-dependent. The CLSM images indicated that a higher cellular uptake (higher green fluorescent intensity) of CG in cells treated with CG-PEG-CSO-SPIONs than with free CG at both tested CG concentrations. This reflects that the CG-loaded NPs were more easily taken up by cells via endocytosis, due to their smaller size (~130 nm), while the hydrophobic character of free CG leads to the formation of large insoluble aggregates in aqueous solutions that are more difficult to be uptaken (endocytosis) by cells [38,39].

### 3.8. Magnetic Targeted Delivery

**Figure 13:**
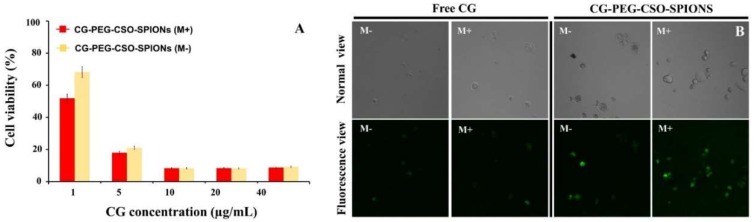
Magnetic targeting delivery analyzed in terms of the (**A**) HT-29 cell viability using the MTT assay (data are shown as the mean ± SD, derived from 3 trials) and (**B**) representative CLSM images of HT-29 cells after incubation with free CG or CG-PEG-CSO-SPIONs at 40 µg CG/mL for 24 h with (M+) or without (M−) an external magnetic field.

To investigate the potential of CG-PEG-CSO-SPIONs for magnetic targeting, HT-29 cells were treated with CG-PEG-CSO-SPIONs at the equivalent concentration of free CG ranging from 1 ug/mL to 40 µg/mL and exposed to an external magnetic field. Whilst the cytotoxicity of CG was enhanced by loading in PEG-CSO-SPIONs (Figure 11 and Figure 12), an ever greater cytotoxicity was observed after applying an external magnetic field (Figure 13). For example, at 1 µg/mL of CG concentration, the viability of HT-29 cells treated with CG-PEG-CSO-SPIONs under a magnetic field was 52%, whereas that without a magnetic field was 68%. Thus, more CG-PEG-CSO-SPIONs were uptaken by cells after application of the external magnetic field (Figure 13B). The CLSM images revealed no difference in the green fluorescent intensity in HT-29 cells treated with free CG with or without an applied magnetic field, and so the cellular uptake of free CG was not affected by the presence or not of the magnetic field. In contrast, the cells treated with CG-PEG-CSO-SPIONs under an applied magnetic field showed a significantly increased green fluorescent intensity compared to those treated without the magnetic field. Thus, the enhanced CG uptake by the HT-29 cells was due to the accumulation of CG-PEG-CSO-SPIONs to the target area by the external magnetic force giving an increased local concentration.

## 4. Conclusions

The PEG-CSO-SPION system was successfully fabricated for loading and delivery of CG to target cancer cells. The optimized CG-PEG-CSO-SPIONs had a spherical shape with a smooth surface and narrow size distribution, and displayed superparamagnetism characteristics. The in vitro release profiles at physiological and tumor extracellular environments indicated the controlled and sustained released manners. The CG-PEG-CSO-SPIONs showed only a low level of non-specific protein (BSA) binding onto their surface and showed a high cytotoxicity on HT-29 cells. For anticancer drug screening, the CG-PEG-CSO-SPIONs should be further tested against other colorectal cancer cells such as Caco-2 and HCT116 cell lines, as well as primary cells of other tissues. With an alternative magnetic field, the temperature of a local environment of a tumor is increased by magnetic heating of SPIONs, resulting in a change the physiology of tumors, leading to the cell death. Therefore, our overall results suggest that CG-PEG-CSO-SPIONs have the potential to be used as a superior drug carrier for targeting to colon cancer cells under magnetic hyperthermia (MHT) condition.

## Figures and Tables

**Table 1:** Factors and responses of CG-PEG-CSO-SPIONs in the PBD.

**Factor**	**Level**	
	**Low (−)**	**High (+)**
X_1_ = CTAB concentration (mg/mL)	2.5	7.5
X_2_ = CSO concentration (mg/mL)	0.1	0.3
X_3_ = PEG concentration (mg/mL)	0.1	0.3
X_4_ = Molecular weight of PEG (Da)	1000	3000
X_5_ = CG concentration (mg/mL)	0.2	0.4
**Response**	**Constraint**	
Y_1_ = Particle size (nm)	Minimize	
Y_2_ = Zeta potential (mV)	≥ ±20 mV	
Y_3_ = LE (%)	Maximize	
Y_4_ = LC (%)	Maximize	

**Table 2:** Factors and observed responses in the PBD.

Run	Factor (X_1–5_)					Response (Y_1–4_)			
	X_1_	X_2_	X_3_	X_4_	X_5_	Y_1_	Y_2_	Y_3_	Y_4_
1	7.5	0.3	0.1	3000	0.4	435 ± 23	25.2 ± 1.1	40.5 ± 3.2	4.7 ± 1.3
2	2.5	0.3	0.3	1000	0.4	512 ± 37	19.3 ± 0.8	33.4 ± 4.6	3.2 ± 0.6
3	7.5	0.1	0.3	3000	0.2	492 ± 17	31.1 ± 0.4	35.4 ± 2.8	4.5 ± 1.6
4	2.5	0.3	0.1	3000	0.4	323 ± 28	20.2 ± 0.7	37.5 ± 1.9	3.8 ± 0.3
5	2.5	0.1	0.3	1000	0.4	387 ± 42	24.2 ± 1.2	45.1 ± 5.2	3.8 ± 1.1
6	2.5	0.1	0.1	3000	0.2	218 ± 18	23.2 ± 1.9	55.1 ± 6.7	5.7 ± 1.9
7	7.5	0.1	0.1	1000	0.4	238 ± 24	33.2 ± 0.2	68.4 ± 2.2	7.1 ± 0.8
8	7.5	0.3	0.1	1000	0.2	365 ± 32	23.2 ± 0.1	54.9 ± 3.6	5.7 ± 2.1
9	7.5	0.3	0.3	1000	0.2	575 ± 11	25.4 ± 0.9	35.9 ± 1.9	3.7 ± 1.4
10	2.5	0.3	0.3	3000	0.2	516 ± 12	19.2 ± 1.4	29.3 ± 3.2	3.1 ± 0.8
11	7.5	0.1	0.3	3000	0.4	497 ± 29	32.9 ± 2.1	32.8 ± 3.8	3.4 ± 0.7
12	2.5	0.1	0.1	1000	0.2	169 ± 16	25.8 ± 1.8	62.4 ± 5.1	6.4 ± 1.2
13 *	5	0.2	0.2	2000	0.3	365 ± 34	28.3 ± 1.5	67.1 ± 2.3	6.9 ± 1.8
14 *	5	0.2	0.2	2000	0.3	372 ± 41	29.6 ± 1.7	68.2 ± 1.6	7.1 ± 1.7
15 *	5	0.2	0.2	2000	0.3	368 ± 37	28.2 ± 0.9	66.4 ± 1.2	6.8 ± 2.2

Notes: * is the center point of the design used for error analysis. Response values are shown as the mean ± SD, derived from three trials.

**Table 3:** Factors and responses of CG-PEG-CSO-SPIONs in the BBD.

**Factor**	**Level**		
	**Low (−)**	**Medium (0)**	**High (+)**
X_1_ = CTAB concentration (mg/mL)	2.5	5.0	7.5
X_2_ = CSO concentration (mg/mL)	0.1	0.2	0.3
X_3_ = PEG concentration (mg/mL)	0.1	0.2	0.3
**Response**	**Constraint**		
Y_1_ = Particle size (nm)	Minimize		
Y_2_ = Zeta potential (mV)	≥ ±20 mV		
Y_3_ = LE (%)	Maximize		
Y_4_ = LC (%)	Maximize		

**Table 4:** Factors and responses of the CG-PEG-CSO-SPIONs in the BBD.

Run	Factor (X_1–3_)			Response (Y_1–4_)			
	X_1_	X_2_	X_3_	Y_1_	Y_2_	Y_3_	Y_4_
1	2.5	0.1	0.2	102 ± 22	20.2 ± 2.3	71.7 ± 3.4	6.5 ± 1.5
2	7.5	0.1	0.2	232 ± 17	31.4 ± 1.7	67.3 ± 5.7	4.3 ± 0.8
3	2.5	0.3	0.2	172 ± 13	27.2 ± 2.5	80.3 ± 3.7	5.5 ± 2.1
4	7.5	0.3	0.2	348 ± 35	33.1 ± 1.6	79.6 ± 2.1	6.2 ± 1.6
5	2.5	0.2	0.1	92 ± 32	20.2 ± 1.9	48.9 ± 5.4	4.8 ± 2.4
6	7.5	0.2	0.1	204 ± 11	29.4 ± 0.8	43.5 ± 4.6	7.7 ± 2.8
7	2.5	0.2	0.3	180 ± 28	23.5 ± 2.9	86.4 ± 3.2	7.4 ± 0.6
8	7.5	0.2	0.3	362 ± 19	30.3 ± 1.1	78.9 ± 7.8	3.1 ± 1.1
9	5	0.1	0.1	125 ± 37	26.1 ± 2.1	45.9 ± 5.2	11.3 ± 0.6
10	5	0.3	0.1	196 ± 18	30.2 ± 1.3	59.1 ± 2.3	12.3 ± 1.3
11	5	0.1	0.3	218 ± 32	25.8 ± 1.5	82.7 ± 5.9	10.6 ± 1.5
12	5	0.3	0.3	334 ± 25	32.1 ± 2.2	85.2 ± 8.3	10.9 ± 2.5
13 *	5	0.2	0.2	188 ± 34	29.4 ± 1.8	52.8 ± 2.5	11.0 ± 1.8
14 *	5	0.2	0.2	191 ± 24	31.3 ± 0.7	55.3 ± 9.2	11.4 ± 0.6
15 *	5	0.2	0.2	185 ± 31	28.3 ± 1.2	53.1 ± 7.3	11.3 ± 1.2

Note: * Center point of the design used for error analysis. Response values are shown as the mean ± SD, derived from three trials.

**Table 5:** Summary results of regression analysis for responses.

Response	F-Value	R^2^	R^2^_adjusted_	R^2^_predicted_	Lack of Fit	Remark
**Response Y_1_ (Particle Size)**						
Linear	81.84	0.9571	0.9454	0.9217	0.0198	-
2FI	68.61	0.9809	0.9666	0.9553	0.0296	-
Quadratic	820.04	0.9993	0.9981	0.9918	0.3893	Suggested
Statistically significant factors for Y_1_ (*p* < 0.05) is X_1_, X_2_ and X_3_						
**Response Y_2_ (zeta potential)**						
Linear	6.29	0.6316	0.5311	0.3324	0.0314	-
2FI	6.24	0.8238	0.6917	0.5719	0.0437	-
Quadratic	125.28	0.9840	0.9552	0.8790	0.7375	Suggested
Statistically significant factors for Y_2_ (*p* < 0.05) is X_1_ and X_2_						
**Response Y_3_ (LE)**						
Linear	10.89	0.7482	0.6795	0.5963	0.0197	-
2FI	4.18	0.7580	0.5765	0.2817	0.0137	-
Quadratic	68.02	0.9919	0.9773	0.8856	0.1982	Suggested
Statistically significant factors for Y_3_ (*p* < 0.05) is X_1_, X_2_ and X_3_						
**Response Y_4_ (LC)**						
Linear	0.11	0.0279	−0.2373	−0.8894	0.0030	-
2FI	0.22	0.1404	−0.5043	−2.7683	0.0022	-
Quadratic	685.94	0.9992	0.9977	0.9959	0.9064	Significant
Statistically significant factors for Y_3_ (*p* < 0.05) is X_1_, X_2_ and X_3_						

Notes: X_1_ = CTAB concentration (mg/mL), X_2_ = CSO concentration (mg/mL), X_3_ = PEG concentration (mg/mL), Y_1_ = particle size (nm), Y_2_ = zeta potential (mV), Y_3_ = LE (%) and Y_4_ = LC (%).

**Table 6:** Optimal calculated variables, and the observed, predicted, and residual values for the responses.

Factor	Optimum	Response	Predicted	Observed	% Error
CTAB (mg/mL)	2.8	Particle size (nm)	133	143	6.8
CSO (mg/mL)	0.10	Zeta potential (mV)	30.6	28.2	8.51
PEG (mg/mL)	0.26	LE (%)	83.3	80.2	3.76
		LC (%)	8.34	7.94	4.79
Criterion for optimization = minimum particle size, ≥ ±20 mV of zeta potential, maximum LE and LCDesirability value = 0.96					

Notes: % Error = [(observed value − predicted value)/observed value] × 100%.
